# Biogenic nanoparticles: a comprehensive perspective in synthesis, characterization, application and its challenges

**DOI:** 10.1186/s43141-020-00081-3

**Published:** 2020-10-26

**Authors:** Sunita Patil, Rajkuberan Chandrasekaran

**Affiliations:** 1 Department of Microbiology, Karpagam Academy of Higher Education, Coimbatore, India; 2 Department of Biotechnology, Sri Krishna College of Arts and Science, Coimbatore, India; 3 Department of Biotechnology, Karpagam Academy of Higher Education, Coimbatore, India

**Keywords:** Metallic nanoparticles, Gold, Silver, Antimicrobial, Anticancer, Iron oxide

## Abstract

**Background:**

Translating the conventional scientific concepts into a new robust invention is a much needed one at a present scenario to develop some novel materials with intriguing properties. Particles in nanoscale exhibit superior activity than their bulk counterpart. This unique feature is intensively utilized in physical, chemical, and biological sectors. Each metal is holding unique optical properties that can be utilized to synthesize metallic nanoparticles. At present, versatile nanoparticles were synthesized through chemical and biological methods.

**Main body of abstract:**

Metallic nanoparticles pose numerous scientific merits and have promising industrial applications. But concerning the pros and cons of metallic nanoparticle synthesis methods, researchers elevate to drive the synthesis process of nanoparticles through the utilization of plant resources as a substitute for use of chemicals and reagents under the theme of green chemistry. These synthesized nanoparticles exhibit superior antimicrobial, anticancer, larvicidal, leishmaniasis, wound healing, antioxidant, and as a sensor. Therefore, the utilization of such conceptualized nanoparticles in treating infectious and environmental applications is a warranted one.

**Conclusion:**

Green chemistry is a keen prudence method, in which bioresources is used as a template for the synthesis of nanoparticles. Therefore, in this review, we exclusively update the context of plant-based metallic nanoparticle synthesis, characterization, and applications in detailed coverage. Hopefully, our review will be modernizing the recent trends going on in metallic nanoparticles synthesis for the blooming research fraternities.

**Graphical abstract:**

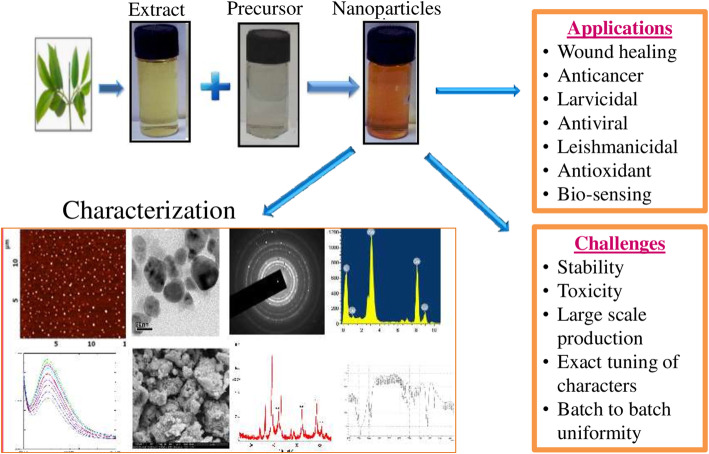

## Highlights

➢ Metallic nanoparticles are a new dimensional form of nanoparticles possessing splendid optical properties.

➢ The synthesis of metallic nanoparticles from plants and its parts is a time deed one.

➢ The characterizations of nanoparticles infer the nano shape and size of the nanoparticles.

➢ Application of nanoparticles in diseases management and environmental sector is emphasized in prolific manner.

➢ Challenges in green synthesis should be resolved and the feasibility of green synthesized nanoparticles in translating into industrial applications must be focused for future medicine.

## Background

Nanotechnology is a new-fangled term that becomes an inescapable part of the modern tool and people are now witnessing the ease of technology in day to day applications [1]. The small-sized nanoparticles (1–100 nm) dominate the entire research globally, due to its stupendous applications in physical, chemical, and biological sciences [2]. Due to intensive and extensive research by the research fraternity, nanotechnology has successfully knocked on the door and a common man at present scenario experiencing the feature of nanotechnology [3].

The delivery address given by Nobel laureate Richard P. Feynman “There’s Plenty of Room at the Bottom” received colossal attention from researchers and there onwards nanotechnology steps ahead and make various revolutionary developments in the field of nanotechnology [4]. When materials are operated at the nanoscale level, the properties of the materials have changed and exhibited tremendous optical, magnetic, and electrical properties. Such kind of unique nano properties is utilized in electronics, batteries, fuel additives, solar cells, catalysts, electrochemical industries, defense, cosmetics, pharmaceuticals, food additives and packaging, agriculture, biosensors, diagnostic imaging, vaccines, antimicrobial and chemotherapy, and drug delivery [5].

Nanoparticles (NP’s) play a decisive role in developing various dimensions of nanomaterials such as carbon nanotubes (CN), metal nanoparticles (MN), ceramic nanoparticles (CN), semiconductor nanoparticles (SN), and polymeric and lipid-based nanoparticles [6]. These kinds of nanoparticles are varying in their morphology, size and shape, and optical properties but excel in various applications in divergent fields [7].

In this review, we conceptualize the green synthesis of metal nanoparticles (MN) synthesis (plants only), characterization, and their biological applications in a lucid approach. Further, we brief the technical challenges of nanoparticles synthesis and its obstacles, toxicity and environmental concern of MN’s, and its perspective for commercialization of MN’s. For the past few decades, numerous reports have been published in view of MN’s synthesis; but addressing the challenges and its associated problems of MNs is very limited. Due to this rationale, we intend to provide a comprehensive review of MN’s. This review will pose a better understanding of the biosynthesis of nanoparticles (NPs) and their applications to the scientific community in a substantial manner.

## Main text

### Traditional nano concepts and its applications

Nanotechnology is not an era of modern science while reverting to history; nanotechnology exists in the history of arts and nature beings. In sculpture, gold and copper are mixed with other substances and reduced in a defined temperature into respective metal ions. This resultant mixture is applied on the surface of the coatings to make a glittering effect. Naturally, NP operates at the nanoscale in various living beings. These natural nanomaterials have the unique property of molecular recognition due to which they can self assemble [8]. The most dynamic example of natural nanoparticles is a nanoscopic wax crystal papillae in the upper side of each epidermal cell of lotus leaf to reduce the contact area of water with leaf. As a result, its scientific basis has opened the possibilities of fabricating superhydrophobic surfaces for a variety of products such as Lotusan® a self-cleaning paint (lotus effect), slippery liquid-infused porous surface (SLIPS) used in refrigeration (nepenthes walls). *Bhasma* is a unique Ayurvedic herbal-mineral-metallic compound in the size of nano dimensions (usually 5–50 nm). These are the products of classical Indian alchemy, the “Ayurveda Rasa Shastra,” used for treating diverse chronic ailments.

In the current epoch, nano-based concepts and applications are again flourishing since the 1990s in all scientific sectors. In particular, the Nanobiotechnology concept started at the beginning of the twentieth century exploring various new avenues in the development of nanomedicine and for developing a sustainable environment.

### Metallic nanoparticles

**Fig. 1:**
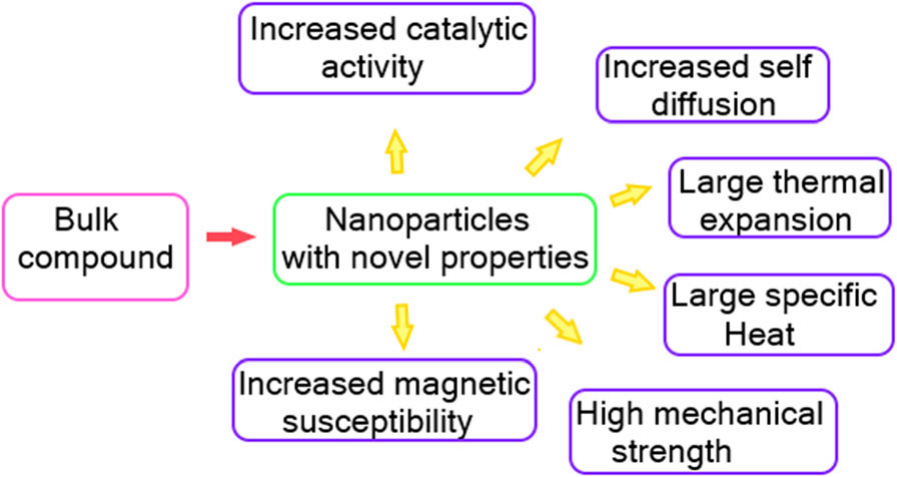
Properties of nonmaterials

Metallic nanoparticles are becoming the limelight of research for scientists and they have proved their competence in various reports addressing the synthesis and applications of versatile inorganic metal nanoparticles (silver, gold, copper, iron, gold, platinum, and palladium) [9] (Fig. 1). The specific properties of metallic nanoparticles are it exhibits prospective optoelectronic and dimensional characteristics superior to their bulk metals [10]. These particular traits render an increase in the surface to volume ratio, reactivity, efficiency, and functional modifications that can tap their potential in diverse applications as multifunctional technical tools [11].

### Nanoparticles synthesis

#### Approaches of nanoparticles synthesis

**Fig. 2:**
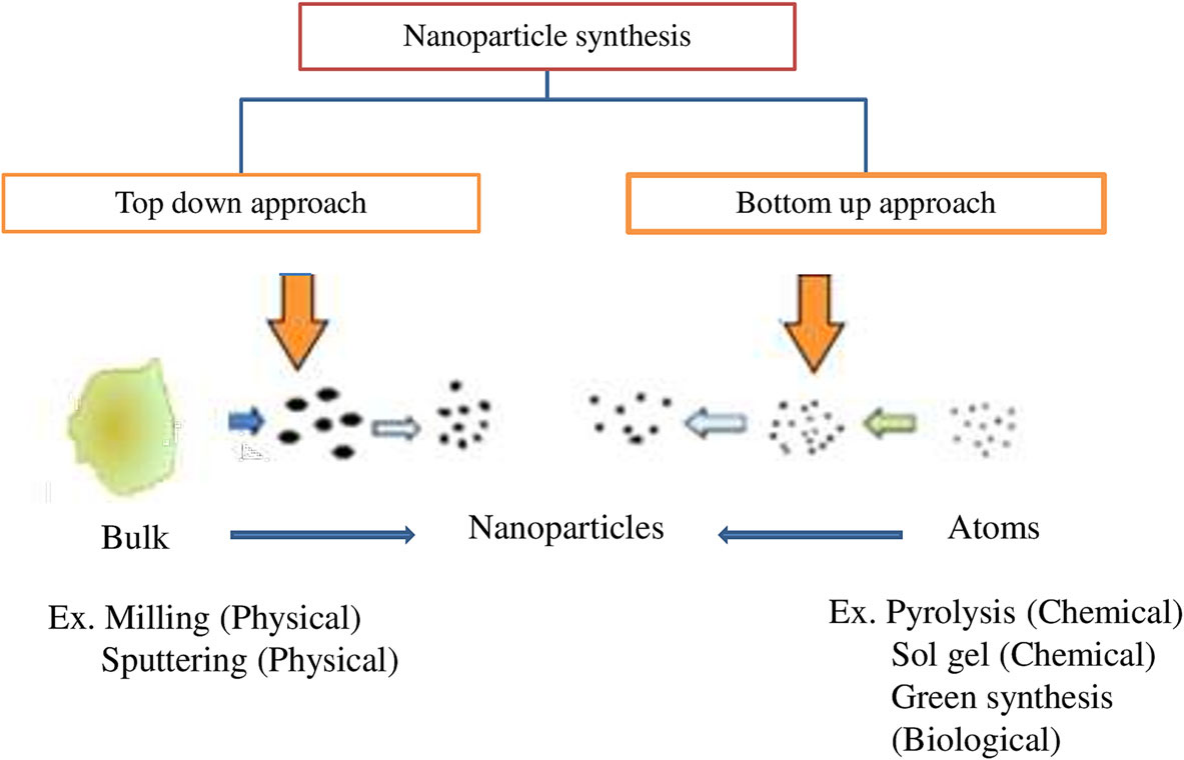
Approaches of nanoparticles synthesis

For fabrication NP’s with the desired shape and size, strikingly there are two classical approaches based on their assemblies followed, either they are top-down and bottom-up approach [12] (Fig. 2). Both these approaches differ in the synthesis principles but finally produce NPs with desired characteristics. In the top-down approach, bulk materials were shattered into the bit to bit pieces leading to the fine generations of NPs. Such kind of NP’s production methods were accomplished by photolithographic techniques, grinding, sputtering, and milling [13]. Each method has its limitations and fine production capacity of NPs. The top-down approach is quite a feasible technique resulting in the production of a large mass of NPs. But the disadvantages associated with top-down are surface imperfection of NPs and in some cases, NP’s may get damaged [14]. The optical and physio-chemical properties of the NPs depend on the surface architecture of NPs; henceforth, top-down approach of NPs synthesis is restricted in some cases of applications.

Another fashionable approach, for NPs production, is the bottom-up approach by coalescence or assembling of atoms by atoms, molecules by molecules, cluster by cluster to generate a diverse range of NP’s [15]. Techniques like self-assembly of monomer/polymer molecules, chemical or electrochemical nanostructural precipitation, sol-gel processing, laser pyrolysis, chemical vapor deposition (CVD), plasma or flame spraying synthesis, and bio-assisted synthesis are employed for the production of NPs [16]. Henceforth, bottom-up is an amenable technique for creating nanoclusters intended for various applications.

### Methods of nanoparticles synthesis

#### Physical methods of nanoparticles synthesis

The synthesis of nanomaterials using physical methods involves deposition, sputtering, ball milling, and plasma-based techniques [16]. The rate of synthesis of metal nanoparticles is very slow in most of these methods. For example, a yield of nanomaterials is 50% or less for ball milling techniques [17]. In the case of sputtering, a big particle size distribution is obtained and only 6–8% of sputtered material is reported to be less than 100 nm. A high-energy consumption is required for laser ablation and plasma techniques. Extensive size distribution, slow production rate, and waste by-products and high consumption of energy make most of the physical methods extremely expensive which cannot be adopted for practical commercial applications [18].

#### Chemical-mediated synthesis of nanoparticles

A variety of chemical methods for nanoparticle synthesis has been put forward and most of them are widely used to synthesize nanostructured materials (e.g., chemical reduction, pyrolysis, sol-gel method, microemulsion, polyol synthesis, hydrothermal synthesis, chemical vapor deposition) [19]. Moreover, employing hazardous chemicals and reagents during the synthesis process and generation of byproducts is lethal to humans and the environment also [20]. Therefore, specifically such kind of NPs is limited for biological applications.

#### Biological-mediated synthesis of nanoparticles

Green nanotechnology is an emerging field to design novel NPs using a green chemistry approach. Biological methods of NPs synthesis provide a new possibility of synthesizing NPs using natural reducing and stabilizing agents. It is an economical and environmentally friendly alternative to chemical and physical approaches with no usage of energy and toxic chemicals.

Biological synthesis of NPs is a bottom-up approach that involves the use of simple unicellular to complex multicellular biological entities like bacteria, fungi, actinomycetes and yeast, algae, and plant materials [21–27]. Microbial-mediated synthesis of nanoparticles is another variant method of producing nanoparticles. In this synthesis method, microbial culture filtrates (extracellular and intracellular) are used as a reducing agent for nanoparticles production. Generically, microbes like bacteria, fungi, yeast, and actinomycetes having the metal-tolerant capability and thrive at utmost environmental conditions [28]. These inherent features are employed by microbes to tolerate, accumulate, and convert metal into respective metal ions. For instance, the first bacterial gold nanoparticles were synthesized from *Bacillus subtilis* [29]. Likewise, the variant face of metallic nanoparticles silver, gold, copper, iron, zinc, platinum, and selenium were synthesized from the bacterium. The common phenomenon in reducing metals into metal ions is by redox reactions through the intracellular/extracellular pathway. At first, the metal is trapped onto the surface of bacterial cells while later, these trapped metals were exclusively reduced into metal ions by the action of enzyme NADH and NADH-dependent nitrate reductase enzymes [30]. These enzymes perform electron shuttle donor processes during synthesizing nanoparticles which are reported in the synthesis of silver nanoparticles from *Bacillus licheniformis* [31].

In fungi, *Fusarium oxysporum* synthesized silver nanoparticles by the action of nitrate reductase and anthraquinones [32]. Conceivably, with the above bacterial- and fungal-mediated synthesis of metallic nanoparticles, it is evident that NADPH nitrate reductase is a major biofactor in the synthesis of metallic nanoparticles.

Though green nanoparticles are a new alternative method for conventional nanoparticles synthesis, but for a nanoparticles synthesis and production, an ease method should be adopted. In-universe, amply bio-resources (plants, microbes) were available. But for synthesis and commercialization perspective, utilization of such bioresources is imperative. In such a case, microbes can be effectively utilized; expensive, but the handling of microbes, scale-up process, molecular mutation, hurdles in mass cultivation, downstream processing, and other factors make a bottleneck for nanoparticles synthesis and application. Henceforth, research should drive lucidly; employing plants as a resource in nanoparticles synthesis is indeed one. Plants bestowed with numerous active constituents phenols, alkaloids, flavonoids, terpenoids, saponins, tannins, polysaccharides, polyphenols vitamins, etc. These constituents were effectively reduced and stabilized the nanoparticles. Moreover, using plants as a resource for synthesis offers advantages such as plant material availability, cost inexpensive, easy scalable for mass production, secondary metabolites, and purgative properties. Proper and optimized use of biological entities for the synthesis of NPs will produce well-characterized and highly stable NPs.

**Fig. 3:**
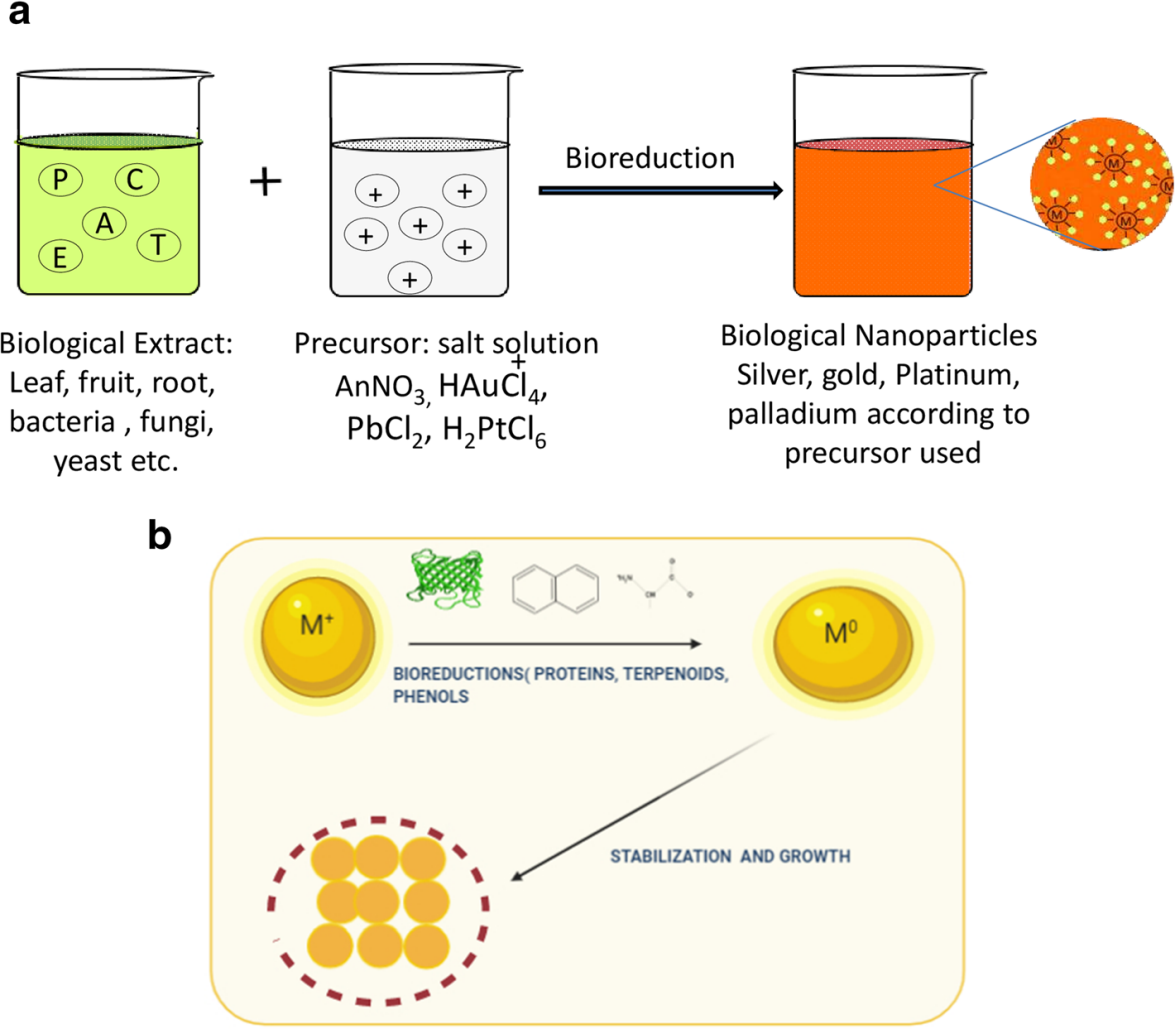
**a** Biological synthesis of nanoparticles. **b** Mechanism of plant-mediated metallic nanoparticles synthesis

Schematically, a generic equation for the NPs synthesis is as follows:

$$ A+B\underset{{}^{\circ}C,T, RPM, pH}{\overset{\varDelta }{\to }}C+D $$ (Fig. 3a, b)

*A* denote plant moieties; *B* denote chemical precursors [salts].

When *A* reacted with *B* [salt] in the presence of heat, temperature, rotation per minute [RPM], and pH, *A* reduce *B* into respective metal ions and by-products. The rate of reduction and generation of NPs is influenced by various factors such as time, temperature, stiochemistric proportion, and pH. The synthesis of metallic nanoparticles is accountable by the action of phytoconstituents present in the plant extracts. Plants endowed with numerous active constituents; these constituents activate the reaction mechanism and synthesize the metallic nanoparticles. Similarly, the synthesis of metallic oxide nanoparticles is the same process but until now a lucid mechanism is not yet been explored [33].

### Technical challenges for nanoparticles synthesis

Generally, the production of NPs with a specific shape, size, and distribution can be achieved by changing the methods of synthesis, the reducing agents, and stabilizers [34]. The data from Table 1 showed how plant extract greatly affects the size and shape of NPs. There are variations in plant extract used and the methodology adopted for the synthesis is important to standardize and optimize the synthesis protocol to get NPs with desired size, shape, and surface charges.

**Table 1:** Effect of biological material, precursor concentration and extract concentration on the morphology of biological nanoparticles

Plant name and part used	Nanoparticles	Precursor	Concentration of plant extract used	Size	Shape	References
*Aloe vera* leaf	Gold and Silver	1 mM HAuCl_4_ 10 mM AgNO_3_	0.5–4 mL 5 mL	5–50 nm	Gold-triangular, spherical Silver-spherical	[35]
*Avena sativa* biomass	Gold	0.1 mM Au(III)	2 mL	5–20 nm	Tetrahedral, deca- hedral, hexagonal, icosahedral multitwinned, irregular shape, and rod shape nanoparticles	[36]
Black tea leaf extract	Gold and Silver	0.01 M HAuCl_4_ AgNO_3_	10 mL	20	spherical	[37]
*Cinnamon zeylanicum* bark powder	Silver	1 mM AgNO_3_	5 mL	31–40	cubic, hexagonal	[38]
*Hibiscus rosa sinensis* leaf	Gold, Silver	0.0005 M HAuCl_4_ 0.0008 M AgNO_3_	5 mL 20 mL	14 13	Gold-triangular, hexagonal, dodecahedral and spherical Silver-spherical	[39]
*Ocimum sanctum* leaf	Gold, Silver	1 mM AuCl_4_ 1 mM AgNO_3_	30 mL 25 mL	30 13	Gold-hexagonal, prism Silver-spherical	[40]
*Parthenium hysterophorus* leaf	Silver	1 mM AgNO_3_	50 mL	50	Irregular	[41]
Pear fruit extract	Gold	2 mM HAuCl_4_	500 mL	200–500	Triangular, hexagonal	[42]
*Tamarindus indica* leaf	Gold	1 mM HAuCl_4_	45 mL	20–40	triangular	[43]
*Garcinia mangostana* pericarp	Gold, Silver	10 mM, HAuCl_4_ 10 mM, AgNO_3_	3 mL	13.65 ± 5.07 to 31.08 ± 3.99	Nanodumbbell shapes	[44]
*Terminalia chebula* Seed	Gold	0.01 M HAuCl_4_	2 mL	6–60	Triangles, pentagons and spheres	[45]
*Cassia tora* leaf	Gold	1 mM HAuCl_4_	100 mL	41-57	spherical	[46]
*Madhuca longifolia* flower	Silver	1 mM AgNO_3_	30 mL	30-50	Spherical and oval	[47]
banana stem	Silver	2 mM AgNO_3_	9 mL	75.50 nm to 1.22 μm	Truncated octahedron, rhomb-dodecahedron, cubic, octahedron and octagon	[48]
Pine cone	Silver	1 mM AgNO_3_	45 mL	1–50	Triangular and hexagonal	[49]
Lycium chinense fruit	Gold, Silver	1 mM HAuCl_4_ 1 mM AgNO_3_	50 mL	20–100 nm, 50–200 nm	Polydispersed, spherical	[50]
*Ocimum sanctum* (tulsi) leaves	Platinum	1mM H_2_PtCl_6_	190 mL	23 nm	Spherical	[51]
*Diopyros kaki* leaf	Platinum	1mM H_2_PtCl_6_	190 mL	2–20 nm	Spheres and plates	[52]
*Curcuma longa* tuber	Palladium	1 mM PdCl_2_	50 mL	15 to 20 nm	Spherical	[53]
*Musa ornate* flower	Iron	5 mM FeSO_4_	10 mL	43.69 nm		[54]
*Calotropis* Gigantean flower	Iron	0.01 M FeNO_3_.9H_2_O	10 mL	50–90 nm	Spherical	[55]
eucalyptus leaf	Iron	0.10 M FeSO_4_	10 mL	20–80 nm	Spherical	[56]
tea powder	Iron	0.1 N Fe(NO_3_)_3_	10 mL	40 to 50 nm	Spherical	[57]
*Platanus orientalis* leaf	Iron oxide	Fe(NO3)3		30–40 nm	Spherical	[58]
*Ficus carica* fruit	Iron oxide	0.14 M FeCl_3_.6H_2_O	10 mL	475 nm	Spherical	[59]
*Conyza Canadensis* leaf	Zinc oxide	0.15 M ZnNO_3_	20 mL in 80 mL	–	Somewhat spherical	[60]
Lemongrass leaves	Zinc oxide	ZnNO_3_	50 mL	85–98 nm	Spherical	[61]
*Hibiscus subdariffa* leaf	Zinc oxide	91 mM ZnC_4_H_6_O_4_	50 mL	12–46 nm	Spherical, dumbbell shaped	[62]
*Costus pictus D*. *Don* leaf	Zinc oxide	0.1 M (Zn(NO_3_)_2_	50 mL	40 nm	Elongated, hexagonal and rodshaped	[63]
*Artemisia abrotanum*	Magnesium oxide	Mg (NO_3_)_2_	90 mL	10 nm	Spherical	[64]
*Trigonella foenum*-*graecum* leaf	Magnesium oxide	5 mM Mg (NO_3_)_2_	150 mL	13 nm	Spherical	[65]
*Punica granatum* Peels	Magnesium oxide	MgSO_4_ 0.1 M	250 mL	50–65 nm		[66]
*Brassica oleracea* flower	Magnesium oxide	MgSO_4_ 0.1 M	250 mL	30–45 nm		

Likewise, the synthesis of NPs using the same plant material showed variation in its characters due to differences in the synthesis method. Table 2 shows a synthesis of metal NPs using *Zingiber officinale* rhizome extract. The researcher used different methods for the preparation of extract, different concentration of precursor, and reducing agent with various temperature and pH. The NPs obtained with these methods are having different features concerning size and shape.

**Table 2:** Synthesis parameters and characters of nanoparticles synthesized using plant *Z*. *officinallae*

Nanoparticles	Concentration of precursor	Concentration of *Z*. *officinallae* extract used	Time for synthesis	Size	Shape	References
Gold, Silver	1 mM, HAuCl_4_ 1 mM AgNO_3_	50 mL	Gold 2.5 h Silver 12 h	Gold 10 nm Silver 30.31 nm	Spherical	[67]
Silver	1 mM AgNO_3_	9 mL	Over night	–		[68]
Gold	1 mM, HAuCl_4_	25 mL	20 min boiling	5 to 15 nm	Spherical	[69]
Silver	1–3 mM AgNO_3_	25 mL	30 min boiling	6 to 20 nm	Spherical	[70]
Gold, Silver	1 mM, HAuCl_4_ 1 mM AgNO_3_	45 mL	10–210 min	20 to 100 nm	Spherical	[71]
Gold	0.2 mM, HAuCl_4_	10 mL	24 h	3.22 nm	Spherical	[72]
Silver	1 mM AgNO_3_	50 mL	1 h	3.1 nm	Spherical	[73]
Silver	1 mM AgNO_3_	20 mL	2 h	10.10–18.33 nm	Spherical tetragonal	[74]

In aspects of large-scale synthesis, among the plant materials, leaves can be extensively used for large-scale synthesis. The plant material (leaves) will be available at all times and all seasons. Moreover, the plants will not be affected by using leaves but using other resources like a flower, fruit, seed, root, and latex will also be meaningful but the volume of materials and it should not affect crop productivity. Moreover, the plant-based nanoparticles are reproducible, stable, and environmentally friendly also. In plant-mediated nanoparticles, various parameters like pH, precursor, and extract concentration, time, and other factors will determine the size of nanoparticles. Since plant constituents were different in species and genera level, so optimization of these parameters will eventually produce nanoparticles with the desired size and shape. Another important concern of nanoparticles is stability. The colloidal stability of nanoparticles is important for long-term application studies. Comparatively, chemical-mediated synthesis of nanoparticles is stable for a long duration; biological synthesis of nanoparticles stability is determined by the capping agents. In a study, silver nanoparticles are synthesized chemically and biologically; the zeta potential of chemical AgNPs is 17.8 mV and biological AgNPs are 15.2 mV [75]. In biological nanoparticles, the stability of the nanoparticles solution is due to the stabilization of the metal particles by the biomolecules. Moreover, the stability of the nanoparticles is determined by pH, surface capping agents, and functionalization techniques.

### Effect of pH

The role of pH during nanoparticle synthesis not only affects size but also the shape of the particle. Yang and Li [76] demonstrated the shape of the product prepared under lower pH was less regular and tend to aggregate. While synthesis of NPs under different pH conditions, the size of particles can be produced with the desired size and shape uniformly [77–79]. The pH causes the local surface of nanoparticles by protonation and deprotonation of molecular atoms in the nucleation and growth stage of NPs [80]. At the alkaline pH range, the NPs forms cluster distribution in the colloidal stage preventing aggregation [81]. Armendari et al. [36] demonstrated the role of pH in gold nanoparticles synthesis and as a result, the synthesized nanoparticles exhibit tetrahedral, decahedral, hexagonal, icosahedral multitwinned, irregular, and rod shape at pH values of 2, 3, and 4. Therefore, formations of truncated octahedron, rhomb-dodecahedron, cubic, octahedron, and octagon structures are thermodynamically favored at the nucleation stage and at the initial growth stage when the particle sizes are not very large [48].

### Effect of precursor and reducing agents concentration

The concentration of reactants like precursors and reducing agents also affect the size of NPs formed. This phenomenon may be due to too many reducing agents bound to the surface of preformed nuclei, which intensifies the secondary reduction of silver ions on the surface of the nuclei. Consequently, the growth rate of NPs is increased, leading to larger NPs. On the other hand, too many reducing agents may enhance the bridging effect among the formed NPs, resulting in the aggregation of NPs. This may be due to too many metal ions absorbed on the surface of preformed nuclei, where the secondary reduction process occurred leading to form larger NPs [82]. Not only size, but the shape of NPs will also get affected (Tables 1 and 2). Chandran et al. [26] and Shankar et al. [83] reported the percentage of triangles formed in the reaction medium as a function of varying amounts of the plant extract reveals that more spherical particles are formed with an increasing amount of extract. So, the optimum concentration of both the reducing agent and precursor is necessary to get the desired nanoparticle size.

### Effect of temperature

As the temperature increases during nanoparticles synthesis, the rate of producing NPs from large to small size is achieved. Generally, high temperature is conducive to nucleation for growth for larger nanoparticles [27, 76, 84, 85]. Low temperature is conducive to growth; however, it is observed that the total reaction rate is increased with the increasing reactive temperature. Temperature exhibits different effects on the size of NPs under sufficient and insufficient quantity of the precursors due to its impressively different influence on the nucleation kinetics constant k1 and growth kinetics constant k2 [86]. As the reaction temperature increases, the reduction rate increases and thus most metal ions are consumed in the formation of nuclei, blocking the secondary reduction process on the surface of the preformed nuclei. Therefore, small and highly dispersed NPs are formed with increased yield [76].

The characteristics NPs synthesized using biological methods are greatly influenced by the incubation time of the reaction medium. The variations in characters during long time incubation may occur due to aggregation or shrinkage of particles; the self-life of particles may affect the potential of particles [87]. Factors governing the nanoparticles were enlisted in Table 3.

**Table 3:** Parameters manipulating the biosynthesis of nanoparticles

Parameters	Effect on biosynthesis of nanoparticles
pH	Variability in size and shape
Reaction temperature	Size, shape, yield, and stability
Reactants concentration	Variability in shape
Reaction time	Increase in reaction time increases the size of metal nanoparticles

### Applications

#### Antimicrobial activity

Development of resistance against antibiotics is threatening the scientific world globally; therefore, it is indeed to develop a pronounced novel material to alleviate against antimicrobial-resistant strains. Since antiquity, metals like copper, silver, iron, gold, magnesium, and other metals are practiced in traditional medicine. Inherently, these metals possess antimicrobial activity [88]; therefore, researchers elicited to make nano-based metallic/metal oxide NPs as an alternative for developing antimicrobial agents.

Metallic NPs generated from plant sources exhibited numerous biocidal activities against Gram-positive, Gram-negative bacterium and eukaryotes [89]. It is also reported that metallic NPs displayed effective inhibitory activity against resistant strains like *Pseudomonas aeruginosa*, ampicillin-resistant *Escherichia coli*, erythromycin-resistant *Streptococcus pyogenes*, methicillin-resistant *Staphylococcus aureus* [MRSA], and vancomycin-resistant *Staphylococcus aureus* [VRSA] [90]. In the present review, we provide the antimicrobial activity of metallic NPs synthesized from plant sources against bacterium and fungi. The antimicrobial activity of NPs depends on the type of metals used, NPs [size, shape, pH, charge, and coating agent], genus, and species [91]. Generally, it is presumed that NPs have a high surface to volume ratio than bulk metallic counterparts, which enable them to easily interact with the cell membrane [92].

Different metallic NPs exert multiple mechanisms to counteract the microbial activity. Numerous reports have postulated the antimicrobial mechanism of NPs but until now a precise mechanism has not yet been justified for the mechanistic action of NPs. Generally, biogenic metallic/metal oxide NPs exert their bactericidal activity by releasing metal ions; interaction with cell membrane leading to damage of cell membrane and thereby formation of pits/gaps in the cell membrane leading to fragmentation of cell membrane [93]. Consequently, NPs interact with sulfur/thiol and phosphorus of proteins or DNA, leading to disruption of the metabolic process [respiratory chain, DNA replication, protein synthesis] and finally cell death [94]. Likewise, NPs exert their bactericidal activity by triggering the production of ROS followed by damaged cell wall integrity caused by phospholipid oxidation and then the internal collapse of proteins/DNA/RNA [95]. Moreover, the antibacterial activity of NPs variably differs from Gram-positive and Gram-negative bacterium due to the presence of a thick peptidoglycan layer which acts as a barrier for penetration of NPs [96].

Fungi cell architecture is made up of well cell membrane and cell wall; cell membrane is made up of phospholipids and cell wall contain mannoproteins, β-1,3-d-glucan and β-1,6-d-glucan proteins, chitin, proteins, lipids, and polysaccharides [chitin, glucan, and mannan or galactomannan] [97]. Antifungal activity of metallic NPs is initiated by interaction with the cell wall and membrane diffusion of metal ions followed by inhibition of β-glucan synthase or on *N*-acetylglucosamine [*N*-acetyl-d-glucose-2-amine] an important component in the cell wall of fungi [98]. Further induction of ROS followed by oxidative stress which eventually interacts with macromolecules [DNA/RNA/Proteins] and leads to cell death [99]. In Table 4, we herein provided the antimicrobial activity of metallic nanoparticles synthesized from plants.

**Table 4:** Antimicrobial activity of biological metal nanoparticles

S. No	NP’s	Source	Microbes	Activity	Ref
1	Tio_2_	*Hibiscus rosa*-*sinensis*	*Vibrio cholerae*, *Pseudomonas aeruginosa and Staphylococcus aureus*	Disc diffusion method	[100]
		*M*. *citrifolia*	*Staphylococcus aureus*, *Escherichia coli*, *Pseudomonas aeruginosa*, *Bacillus subtilis*	Agar well diffusion method	[101]
			*Candida albicans Aspergillus niger*		
2.	Pt	*Taraxacum laevigatum*	*Bacillus subtilis*, *Pseudomonas aeruginosa*	MHA well diffusion method	[102]
3.	Pd	*Garcinia Pedunculata*	*Cronobacter sakazakii*	Agar well diffusion method, MIC and MBC	[103]
4.	Se	*Emblica officinalis*	*Escherichia coli*, *Listeria monocytogenes*, *Staphylococcus aureus* , *Enterococcus faecalis*	Micro well dilution method	[104]
			*Aspergillus brasiliensis* , *A*. *flavus* , *A*. *oryzae*, *A*. *ochraceus*, *Fusarium anthophilum*, *Rhizopus stolonifer*		
5.	Ni	*Monsonia burkeana*	*Escherichia coli*, *Pseudomonas aeruginosa*	Broth dilution method	[105]
6.	Iron oxide	*Acacia nilotica*	*Escherichia coli*, *Marsa*, *Salmonella*, *Staphylococcus aureus*	Gel diffusion assay	[106]
			*Candida*		
7.	Zinc oxide	*Albizia lebbeck*	*Bacillus cereus*, *Staphylococcus aureus*, *Escherichia coli*, *Klebsiella pneumonia*, *Salmonella typhi*	Disc diffusion method	[107]
8.	CuONPs	*Syzygium alternifolium (Wt*.*) Walp*	*Alternaria solani*, *Aspergillus flavus*, *Aspergillus niger*, *Penicillium chrysogenum*, *and Trichoderma harzianum*	Disc diffusion assay	[108]
9	Au	*Ziziphus zizyphus*	*Escherichia coli*	Radial diffusion assay	[109]
			*C*. *albicans*	Micro dilution plate assay	
10.	Ag	*Erythrina suberosa (Roxb*.*)*	*Bacillus subtilis*, *Staphylococcus aureus*, *Pseudomonas aeruginosa*, *Escherichia coli*	agar cup and micro broth dilution method	[110]
			*C*. *albicans*, *C*. *kruseii*, *T*. *mentagrophytes*, *C*. *viswanathii*	broth dilution method	

#### Anti-inflammatory activity of metallic NPs

Inflammation is a localized phenomenon that occurs as a result of injury, infection and stress by multiple mechanisms like recruitment of macrophages, killer cells cytokines like IL-1, IL-1β, and TNF-α to the desired site and develops the onset of inflammation [111]. Conventionally, steroidal and nonsteroidal anti-inflammatory drugs are administered for inflammation but the side effect exerted by the drugs had an adverse effect [112]. Nano-based herbal formulation is proved as a pioneer in developing anti-inflammatory drugs. Numerous articles emphasize the metallic NPs synthesized from plant extracts endowed with anti-inflammatory properties. Recently, a study concluded that silver NPs generated from *Selaginella myosurus* demonstrated the anti-inflammatory potential under in vivo and in vitro conditions. The study implied that AgNPs can be able to inhibit the protein denaturation, which is an important phenomenon in inflammation in the Carrageenan-induced rat hind paw edema model AgNPs that interferes with the release of acute inflammatory mediators [histamines, serotonin, kinins, prostaglandins, and cyclooxygenase products] and antagonizes their action [113]. Similarly, gold NPs synthesized from *Prunus serrulata* was assayed against LPS-induced RAW264.7 macrophage [114]. The results depicted that AuNPs suppressed the production of inflammatory mediators and pro-inflammatory cytokines in LPS-induced in RAW264.7 cells by inhibiting NF-jB activation. Nagajyothi et al. [115] demonstrated that zinc oxide NPs from *Polygala tenuifolia* root extract displayed promising anti-inflammatory activity by inhibiting the expressions of proteins iNOS, COX-2, IL-1b, IL-6, and TNF-α. Recently in an investigation, anti-inflammatory activities of selenium NPs coated with polysaccharide of *Ulva lactua* effectively inhibited the NF-κB protein in DSS-induced colitis mice [116]. The above-mentioned reports suggest that green synthesized metallic NPs can be able to minimize the inflammation with greater efficiency, by blocking pro-inflammatory cytokines, ROS scavenging mechanisms, and inhibiting the NF-κB and COX-2 pathways.

#### Wound healing properties

A wound is defined as sharp injuries to skin tissues where the dermal layers are cut, punctured, or torn due to response to stimuli or trauma [117]. Generally, wounds are classified into two types, namely acute and chronic wounds, based on healing time and other complications [30]. Healing of wound is a phenomenal process in which various factors intricate each other between various cell types, coagulation factors, connective tissue, growth factors, cytokines, and the vascular system. There are four phases of the wound healing process: (i) hemostasis phase, (ii) inflammatory phase, (iii) proliferative phase, and (iv) maturation phase. These four phases are a complex process and coordinately function altogether to heal the wound. Failure in any phase led to chronic wound and its complications are severe [118]. Moreover, other factors lead to impaired wound healing, such as diabetes, obesity, malnutrition, medication, and lifestyle habits, including excessive alcohol intake and smoking [119]. The current therapies involve the use of hyperbaric oxygen therapy, negative pressure wound therapy, bioengineered cell construct, dressing materials, and vascular surgery. Besides medications like steroidal drugs (glucocorticoid drugs), nonsteroidal drugs (ibuprofen, naproxen, rofecoxib, and celecoxib) and chemotherapeutic drugs (bevacizumab, lenvatinib, cabozantinib, brivanib, refametinib, and everolimus) are commonly practiced. But these drugs are all associated with various side effects which limit the usage [120].

In traditional medicine, plant extracts, honey, maggots, propolis, and larvae are a fascinating alternative for wound therapy [117]. But howsoever, with the advent of science and technology, researchers drive their focus in wound therapy by involving herbal extracts with nano concepts to address the specificity and complexity associated with wounds. Nano-based approaches for wound therapy is comprised of two groups; in one group, nanomaterials (metals, metal oxide, metalloid) acts as a drug for wound healing while the latter nanomaterials (growth factors, nucleic acids, small molecules) act as vehicles/delivery agent to repair wound [118].

Recently, silver NPs generated from *Lindera strychnifolia* claimed to have wound healing property determined by the cell scratch method on NIH3T3 cells [121]. Garg et al. [122] reported the synthesis of silver NPs from the root extract of *Arnebia nobilis* and formulated with hydrogel and applied in albino rats. The results demonstrated that formulated silver NPs exhibit splendid antibacterial and healing activity. Interestingly, *Coleus forskohlii* root extract generated silver and gold NPs displayed prominent wound healing activity in excision wounds in albino Wistar male rats [123]. Moreover, NPs do not exert a toxic effect on the animals and stimulate re-epithelialization of cells in a shorter period. Shankar et al. [124] fabricated copper oxide NPs from *Ficus religosa* leaf extract. The synthesized copper oxide NPs rendered superior wound healing activity and upregulated major 60, 47, 32, 26, and 25 kDa proteins which play an important role in the different phases of wound repair, wound contraction, and re-epithelialization process. Moniri et al. [125] reported the synthesis of magnetic NPs (Fe_3_O_4_) from *Aloe vera* extract and impregnated the Fe_3_O_4_ NPs in bacterial nano cellulose (BNC) to form a nanocomposite BNC/Fe_3_O_4_. Under in vitro *conditions*, BNC/Fe_3_O_4_ nanocomposite exhibited wound healing activity in HDF cells by scratch assay. Further, BNC/Fe_3_O_4_ nanocomposite triggers the expression of TGF-β1, CTNNB1, MMP2, MMP9, WNT4 and downregulate the expression miR-29b and miR-29c gene. These genes play a pivotal role in the wound healing process and henceforth the nanocomposite is a prominent agent in wound healing. Shao et al. [126] reported that *Barleria gibsoni* leaf extract-mediated zinc oxide NPs ameliorated the wound healing effectively in male albino Wistar rats. Likewise, *Origanum vulgare*-mediated titanium dioxide NPs improve the healing efficacy in an excised wound in male albino Wistar rats [127]. The underlying mechanism behind the wound healing efficacy is not yet clearly understood. But regarding the literature report, we can plausibly affirm that the wound healing reaction is initiated by inhibiting the proliferation of the microbial population. Further, inorganic metallic NPs induce ROS and activate angiogenesis by downregulating 38MAPK/Akt/eNOS-dependent pathway and upregulating key angiogenesis growth factors like vascular endothelial growth factor [VEGF] and fibroblast growth factor [FGF] to accelerate wound healing. Henceforth, it is very essential to understand the molecular mechanism of inorganic metallic NPs-mediated wound healing process for developing metallic NPs as an alternative for wound treatment. Therefore, it is indeed to carry out extensive research to determine the effect of metallic NPs in differing phases of wound healing, toxicity, and biocompatibility to develop NPs as therapeutic potential.

#### Anticancer activity of metallic nanoparticles

Cancer is a dreadful global disease causing major health problems and mortality, accounting for 8.8 million deaths worldwide in 2015 [128]. Metallic NPs have been studied for their novel biological activity to induce autophagy and promote cell death. Additionally, biological metallic NPs are cytotoxic agents to fight against various types of cancer. Some recent in vitro anticancer studies of biological NPs are enlisted in Table 5.

**Table 5:** Anticancer activity of biological metal nanoparticles

Biological agent used for synthesis	Nanoparticle	Cell line	IC_50_ value	Reference
*Marsilea quadrifolia*	Silver	Human ovarian teratocarcinoma (PA-1) and lung adenocarcinoma (A549)	45.88 μg/mL and 52.015 μg/mL	[25]
*Morinda pubescens*	Silver	HEP G2 (Human Epithelium cells of liver cancer	937 μg/mL	[129]
*Nepeta deflersiana*	Silver	Human cervical cancer cells (HeLa)	5 μg/mL	[130]
*Murraya koenigii*	Silver	HT-29 colon cancer	26.05 μg/mL	[131]
*Cyanobacterium Nostoc* sp. strain HKAR-2.	Silver	MCF-7 cells	27.5 μg/mL.	[132]
*Piper nigrum*	Silver	MCF-7 and Hep-2 cells	52 mL, 54 μg/mL	[133]
*Ficus religiosa*	Silver	A549 and Hep2 cells	1.9 μg/mL and 1.6 μg/mL	[134]
*Solanum xanthocarpum*	Gold	HCT 15 human colon cancer	–	[135]
*Streptomyces* sp.	Gold	HeLa	350 μg/mL	[136]
*Evolvulus alsinoides*	Palladium	Human ovarian cancer A2780 cells	–	[137]
*Gloriosa superb*	Platinum and palladium	MCF-7 (human breast adenocarcinoma	49.65 ± 1.99% and 36.26 ± 0.91%	[138]
*Barleria prionitis*	Platinum Paladium	Human breast adenocarcinoma (MCF-7)	–	[139]
*Sargassum muticum*	Iron oxide nanoparticles	HeLa cells, MCF-7 cells, HepG2 cells, and human cell lines for leukemia Jurkat	1.1 μg/mL (HepG2), 18.75 ± 2.1 μg/mL (MCF-7), 12.5 ± 1.7 μg/mL (HeLa), and 6.4 ± 2.3 μg/mL (Jurkat)	[140]
*Albizia lebbeck*	Zinc oxide	MDA-MB 231 and MCF-7	48.5, 48.7, and 60.2 μg/mL	[107]
*Costus pictus D*. *Don medicinal*	Zinc oxide	DLA	–	[63]
black bean extract	Copper oxide	HeLa	–	[115]
*Ficus religiosa*	Copper oxide	A549 cells	200 μg/mL	[127]

**Fig. 4:**
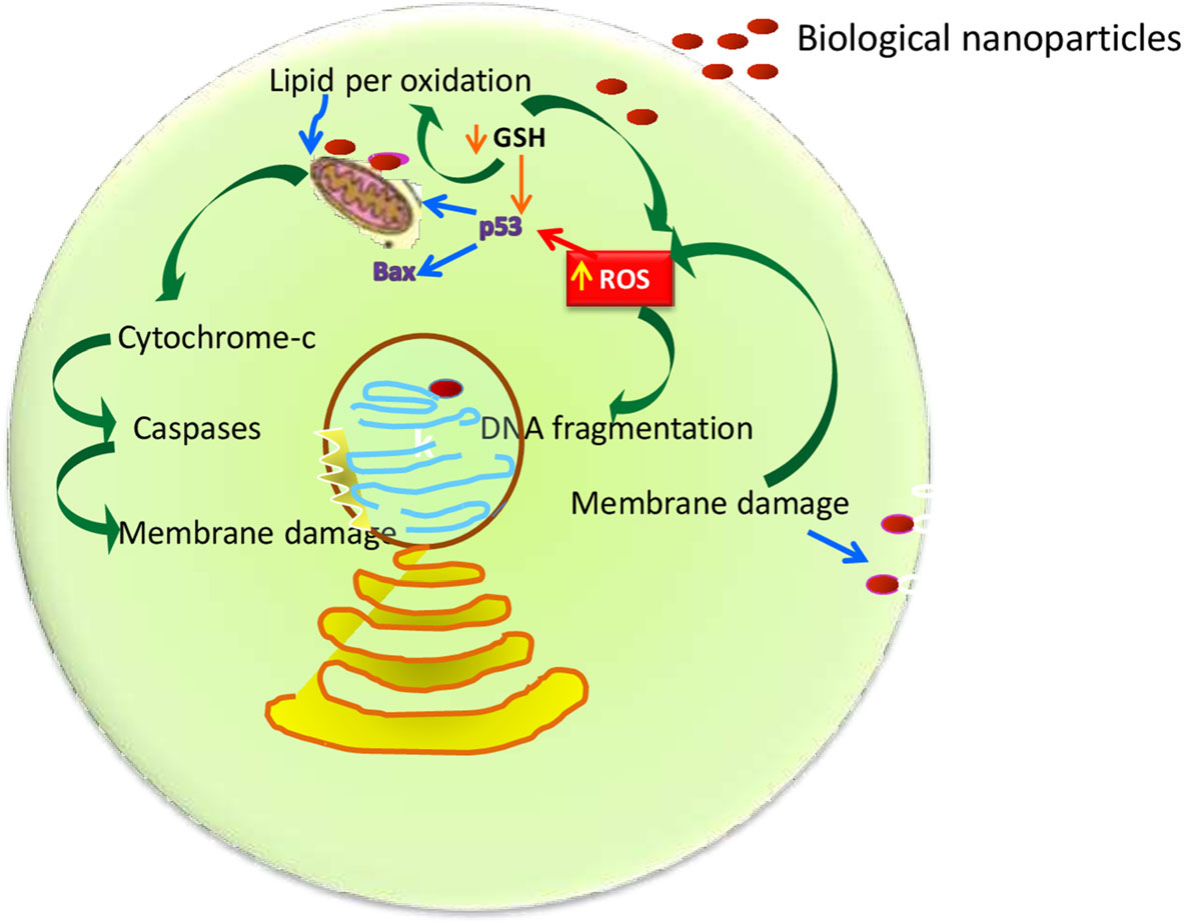
Cytotoxic mechanism of biological nanoparticles

There are three proposed mechanisms for the anticancer activity of biological NPs. Firstly, the apoptotic pathway, which depends on an increased level of ROS which leads to oxidative stress and DNA fragmentation in the cancerous cell [141]. Secondly, interference of proteins/DNA, resulting in cell chemistry functions. Thirdly, the interaction of biological NPs to cell membranes makes changes in the cell permeability and mitochondrial dysfunction [142, 143]. Kim et al. [144] demonstrated that the activation of p38 MAPK and Caspase-3 at gene and protein expression levels results in response to nanoparticles. The general mechanism for anticancer activity biological NPs concluded from various studies is given in Fig. 4

The cytotoxic effect of silver NPs synthesized from *Panax ginseng* fresh leaves (PgAgNPs) exhibited oxidative stress in A549, MCF7, and HepG2 cancer cell lines [145]. PgAgNPs inhibits the epidermal growth factor (EGF) and enhances migration, with decreased mRNA levels and phosphorylation of EGF receptors in A549 cells. Moreover, it modified the morphology of the cell nucleus and increases apoptosis percentage; this effect was linked to the stimulation of the p38 MAPK/p53 pathway. Therefore, up/downregulation of the EGFR/p38 MAPK/p53 pathway might be the possible mechanism of its anti cancer activity by PgAgNPs.

He et al [146] demonstrated in vivo and in vitro cytotoxicity of longan peel powder mediated silver NPs in H1299 cells and in the mouse model. The antagonistic effect of AgNPs was due to the inhibition of NF-κB activity and decrease the expression of Bcl-2/caspase-3 and increase the survivin expression. The apoptotic effect of *Ficus religiosa* leaf extract-mediated copper oxide NPs induce the generation of reactive oxygen species (ROS) involving the disruption of mitochondrial membrane potential [Δψm] in A549 cells [147].

The gold NPs synthesized from *Scutellaria barbata* treated in pancreatic cancer cell lines (PANC-1) demonstrated an upregulated expression level of Caspase 3, Caspase 9, Bax and downregulated the expression of Bid and Bcl-2 [148]. The dose-dependent cytotoxicity of the PdNPs synthesized from *Evolvulus alsinoides* spur the production of (ROS) generation, followed by autophagy and impairment of mitochondrial membrane potential (MMP) [137].

The use of biological NPs is a blooming field in cancer therapy due to their small size and large surface area enables efficient drug delivery, tumor specificity, and promising activity. Now, it is important to conduct research using in vivo models for extending the in vitro findings and to elucidate the mechanisms of biological metallic nanoparticles for the advancement of anticancer therapy.

### Larvicidal activity of nanoparticles

Vector-borne diseases are caused by bacteria, parasites, and viruses that are transmitted by vectors such as mosquitoes, ticks, sandflies, flies, and fleas. Notably, mosquitoes *Aedes aegypti*, *Aedes albopictus*, *Culex quiquefasciatus*, and *Anopheles stephensi* are the vectors that transmit diseases like dengue fever, chikungunya, malaria, yellow fever, lymphatic filariasis, Japanese encephalitis, and West Nile fever [149]. The prevalence of vector-borne diseases in India is very high due to favorable climatic conditions transmitting the diseases exponentially. Governmental agencies framework a layout Pest management Program to control and mitigate the mosquito population through a comprehensive approach [150]. Various strategies have been developed to control mosquito larvae and adult but the eradication of mosquitoes completely is a bottleneck factor for researchers. Larvicides and adulticides are utilized routinely to disrupt the growth of mosquitoes but adulticides are less effective technique in mosquito control [151]. Larvicides are an excellent agent that promotes the disruption of larvae at their breeding sites drastically. Conventionally, larvicides like synthetic insecticide dichlorodiphenyltrichloroethane [(DDT), organochlorines, pyrethroids, and pyrethrins] carbamates and organophosphates, organochlorine cyclodiene and phenylpyrazoles gave positive results and effective to all kinds of mosquitoes at different stages [152]. But howsoever, due to massive usage, mosquitoes start to build up resistance against insecticide. Moreover, insecticides also pose unwanted toxicity to non-target organisms, human beings, and the environment. Considering the pitfalls associated with insecticide, early researchers drive their focus to develop botanical insecticides from plant sources. As a result, plants *Azadirachta indica*, *Nicotiana tabacum*, *Ocimum basilicum*, *Cinnamomum osmophloeum* and plant bases oils have proved the potential as larvicides [153]. At the present scenario, numerous plants and their derivatives have been excelled as a botanical insecticide and some of them are commercialized. As the progress of science and technology, researchers drive their research by formulating nano formulated herbal drugs. It is very indeed at this juncture by combining plants and its products in the nano module to counteract the larvicidal populations.

Table 6 sums up the plant-mediated metallic nanoparticles and their larvicidal activity. The NPs exhibited antagonistic activity against the different instar larvae of mosquitoes. Plant-mediated metallic nanoparticles displayed prominent activity in a dose-dependent manner. Moreover, the effect of larvicidal activity of NPs is directly proportional to the genus and species of mosquitoes, larvae stages, and plant moieties that coat the NPs, size, shape, and charge [154]. All these factors govern the larvicidal efficacy of NPs. The widely accepted precise mechanism behind the NPs toxicity is that they induce oxidative stress in tissues of arthropods. Another study concludes that NPs exert their toxicity by penetrating the cavity of the exoskeleton followed by interaction with sulfur from proteins or phosphorous from DNA leading to rapid denaturation of enzymes and organelles [155, 156]. Jiang et al. [157] also reported that decrease in membrane permeability and disturbance in proton motive force may also lead to impairment of cellular function and death. Subsequently, to shed light on the consequences of exposed NPs, Kalimuthu et al. [158] reported that *Hedychium coronarium* J. Koenig rhizome-synthesized Ag NPs damaged the midgut epithelial cells of *A*. *aegypti* revealed by histopathological study. Sundararajan and Kumari [159] observed that *Artemisia vulgaris* L. mediate gold NPs damage the midgut, epithelial cell, and cortex of *A*. *aegypti* with the accumulation of Au inside the midgut region. In the above-reported studies, accumulation of metals in the thorax and abdomen is a common phenomenon that occurred; this accumulation leads to various complications like ROS generation and cell death.

**Table 6:** Larvicidal activity of metallic nanoparticles synthesized from plant sources

S. No	NPs	Plant	Species	LC_50_	Ref
1.	Ag	*Curcuma zedoaria*	*Culex quinquefasciatus*	0.57 ppm	[160]
2.		*Holarrhena* *antidysenterica (L*.*) Wall*.	*Aedes aegypti ; Culex quinquefasciatus*	5.53 ppm; 9.3 ppm	[161]
3.	Au	*Artemisia vulgaris L*.	*Aedes aegypti*	62.47 ppm	[159]
4.		*Chloroxylon swietenia DC*	*Aedes aegypti* *Anopheles stephensi*	0.423 ppm 0.602 ppm	[162]
5.	Cu	*Tridax procumbens*	*Aedes aegypti*	4.209 ppm	[163]
6.	CuO	*Artocarpus heterophyllus*	*Aedes aegypti*	5.08 ppm	[164]
7.	ZnO	*Scadoxus multiflorus*	*Aedes aegypti*	34.04 ppm	[165]
8.	ZnO	*Momordica charantia*	*Anopheles stephensi* *Culex quinquefasciatus*	5.42 ppm 4.87 ppm	[166]
9.	Fe	*Ficus natalensis*	*Aedes aegypti* *Anopheles stephensi* *Culex quinquefasciatus*	35.9 ppm	[167]
10.	Ni	*Aegle marmelos*	*Aedes aegypti* *Anopheles stephensi* *Culex quinquefasciatus*	534.83 ppm 595.23 ppm 520.83 ppm	[168]
11.	Pd	*Cocos nucifera*	*Aedes aegypti*	259.24 ppm	[169]
12.	Se	*Clausena dentata*	*Aedes aegypti* *Anopheles stephensi* *Culex quinquefasciatus*	104.13 ppm 240.71 ppm 99.60 ppm	[170]
13.	Tio2	*Morinda citrifolia*	*Aedes aegypti* *Anopheles stephensi* *Culex quinquefasciatus*	18.62 ppm 5.71 ppm 33.69 ppm	[171]
14.		*Mangifera indica*	*Anopheles subpictus* *Culex quinquefasciatus*	7.72 ppm 8.10 ppm	[172]

### Antiviral activity of metallic nanoparticles

Another important research to be addressed by the researchers is the antiviral properties of green nanomaterials. Viruses are one of the contagious agents causing various diseases in humans, plants, and animals. In particular, the severity of viral diseases in human is panic and cause detrimental effects in human beings [173]. Notably, viruses like influenza, hepatitis, herpes simplex virus [HSV], and human immunodeficiency virus [HIV] are life-threatening to humans. These viruses cause pathologically complicated diseases and if left untreated or vaccinated it will worsen or in some cases, death may occur [174]. In the present scenario, numerous antibiotics identified from plant and microbial sources have been developed and formulated and commercialized as antiviral agents [175]. These drugs exert their mechanism effectively, but prolonged administration of drugs causes the virus to build up resistance mechanisms [176]. In the crusade to develop fabricated nano drugs for antiviral therapy, plant-based metallic nanoparticles have open up the potential to combat viral diseases.

Among the metallic nanoparticles, silver NPs are ranked top in antiviral NPs. This is due to the fact that application of silver as an eye solution for the treatment of conjunctivitis [177]. Later, silver NPs synthesized using chemical methods were evaluated against HIV, herpes, Influenza, and hepatitis virus [178]. These results were clinically significant and pave the way for developing NPs as antiviral agents. Despite the facts, concerning the toxicity of metallic nanoparticles through the chemical methods, an alternative strategy should be adopted. Recently, Haggag et al. [179] postulated the green synthesized silver NPs of *Lampranthus coccineus* and *Malephora lutea* and performed splendid antiviral activity against HSV-1, HAV-10, and CoxB4 viruses. The antiviral activity of silver NPs can plausibly confirm that silver NPs bind with viral envelope glycoprotein and inhibit the process. Silver NPs integrate with the viral genome and also inhibit viral replication [178]. Though there are numerous reports on the antiviral activity of metallic nanoparticles synthesized by chemical methods, but until now, there are no scientific reports related to the antiviral activity of plant-mediated metallic nanoparticles except silver. Hence, it is indeed to explore the possibilities of plant metallic nanoparticles to study the antiviral activity.

### Leishmanicidal activity of nanoparticles

Leishmaniasis is another important life-threatening disease caused by the parasite Leishmania transmitted by the sandfly Phlebotomus species [180]. Pathologically, Leishmaniasis can be classified based on severity and intensity as visceral, cutaneous, and postkalaazar dermal leishmaniasis, mucocutaneous leishmaniasis [181]. The therapeutic efficacy of Leishmaniasis has relied on the utilization of antileishmania drugs, but some drugs suffer from resistance mechanisms like increased efflux mechanism, decreased drug concentration inside the parasite, inhibition of drug activation, and inactivation of active drug which hampers the drug activity [182].

Naturally, plants bear the active ingredients to perform antagonistic activity against bacterial and viral infections. Ullah et al. [183] synthesized silver NPs through the chemical and biological method from the aqueous extract of *Teucrium stocksianum* and evaluated for antileishmanicidal activity. The study outputs that both chemical and biogenic NPs demonstrated strong antagonistic assay against *Leishmania infantum* promastigotes with IC_50_ value 30.71 ± 1.91 μg/mL for biogenic AgNPs and 51.23 ± 2.20 μg/mL of chemically synthesized silver NPs. Moreover, the infectivity of Leishmania parasites (treated with chemical and biogenic AgNPs) on macrophages cells was observed by Giemsa staining in the infected macrophages. By MTT assay, the study resulted that chemical AgNPs exert high toxicity while compared with biogenic AgNPs. Ahmad et al. [184] reported the synthesis of gold NPs from an extract of *Maytenus royleanus* and evaluated against *Leishmania tropica* promastigotes. The in-vitro assay resulted that AuNPs drastically reduced the viability of *L*. *tropica* in a shorter period. Likewise, ZnO NPs were synthesized from *Mirabilis jalapa* leaf extract were shown to eradicate the viability of the Leishmania parasite and cause a lethal toxic effect at a concentration of 0.5 M [185]. Similarly, *Rosmarinus officinalis*-mediated iron oxide NPs effectively inhibited the viability of *L*. *major* in a dose-dependent manner with an LC_50_ value of 350 μg/mL [186]. The plausible mechanism antileishmanicidal activity of NPs is hypothesized to be NPs binds with the cell membrane and cause intracellular damage (mitochondria) and inhibition of enzyme synthesis which consequently leads to high-level production of ROS and trigger apoptosis.

### Antioxidant activity

Free radicals are highly reactive unstable atoms or molecules with outermost unpaired electrons generated by reactive oxygen species (ROS). These are responsible for the number of human diseases like atherosclerosis and cancer including brain damage and chronic complications in the physiological system. The electronic configuration of metallic nanoparticles is ready to accept or donate an electron to quench free radicals [187]. Recent reports prescribe that biological NPs synthesized from plants such as copper [IC_50_ 500 μg/mL], copper oxide, gold [IC_50_ 50 μg/mL], silver [IC_50_ 73.27 μg/mL], magnesium oxide [IC_50_ 4.73 μg/mL], and zinc oxide [IC_50_ 127.74 μg/mL] [30, 68, 72, 188–190] shown a radical scavenging activity with significant IC_50_ value. Antioxidant activity of *Albizia lebbeck* stem bark extract-mediated ZnO NPs was carried out using H_2_O_2_ free radical scavenging assay which revealed higher IC_50_ values of 48.5, 48.7, and 60.2 μg/mL for 0.1 M, 0.05 M, and 0.01 M ZnO NPs, respectively [111]. The result obtained from the antioxidant activity study of *Artemisia abrotanum* herb and MgO NPs synthesized using the *A*. *abrotanum* indicated the high antioxidant activity shown by MgO NPs [4.73 μg/mL] as compared to the herb [6.28 μg/mL] itself. Most of the studies are based on 1,1-diphenyl-2-picrylhydrazyl (DPPH) assay while some studies also include nitric oxide, hydrogen peroxide, superoxide, and reducing power assays. The method of synthesis, reducing biological material used for synthesis, and capping agent acquired by nanoparticle play an important role in the antioxidant activity of that nanoparticle [191].

### Toxicity

As the field of nanotechnology began to grow, the potential toxicity of these novel materials is unveiling after its use. Scientists and toxicologists were involved in the safety and evaluation of NPs but very few reports are available on the green NPs toxicity [192]. *Butea monosperma*-mediated silver NPs had shown a therapeutic index of 3.77 when tested over 24 h on human myeloid leukemia (KG-1A) cells and in human peripheral blood mononuclear cells (PBMC) [193]. Biological metallic NPs synthesized from *Abutilon inducum*, *Butea monosperma*, *Gossypium hirsutum*, *Indoneesiella echioides*, and *Melia azedarach* are among the potential anti-cancer agents with an acceptable therapeutic index as their therapeutic index values were > 2.0 when tested on both cancer cells and normal human cells [194].

Silver NPs synthesized rapidly using sulfated polysaccharide extract from *Sargassum siliquosum*, a brown alga when given up to 2000 mg/kg did not cause mortality to rats but caused minimal elevation of serum creatinine and blood urea nitrogen [195]. However, silver NPs synthesized using *Ficus religiosa* leaf extract when administrated in rats revealed a significant increase in serum levels of AST, ALT, and LDH, TNF-a and IL-6 on day 29. These levels reverted to normal at the end of the washout period on day 89. ICP-OES analysis revealed the accumulation of silver in the liver, brain, and lungs on day 29 with the respective concentrations of 4.77, 3.94, and 3.043 μg/g tissue. However, complete elimination of silver was observed on day 89. Histological analysis performed in vital organs indicated pathological changes only in the liver which was also normalized after 89 days [136].

Rheder et al. [196] demonstrated the importance of performing toxicological and ecotoxicological evaluations of new NPs, since although they may provide the desired bactericidal and anticancer effect, their toxicity could threaten the survival of organisms. *Althaea officinalis*-mediated silver NPs cause oxidative stress followed by the increase in CAT and decrease in GPx and GST even in the lower concentration and in almost all tissues. In this circumstance, it seems to reflect an aggravation status due to reduced cell protection ability to protect fish against ROS under stress conditions caused by these NPs. Olive, mulberry, and fig-mediated silver and sulfur have a significant effect on larvae, pupae, and adult mortality and they have decreased larval longevity significantly [197]. Sengottaiyan et al. [198] demonstrated that the *Solanum nigrum* phyto-synthesized silver NPs significantly improved the bodyweight loss in diabetic rats. It also retrieved the total cholesterol and triglyceride levels compared to the normal group for 21 days of administration. While Vasanth and Kurian [199] studied nephrotoxicity of silver NPs prepared by chemical and green route [aqueous extract of *Desmodium gangeticum* root] in rat, proximal epithelial cell lines and renal mitochondria were evaluated by oral administration of silver NPs [100 mg/kg] to the Wistar rats. After 15 days, significant changes in the renal architecture were observed in both receiving rats, supported by the urine and blood chemistry data. Further, exposure toward renal epithelial cells and renal mitochondria also confirm the toxic similarities between the silver NPs synthesized from two routes.

Green-synthesized gold NPs from *Curcuma mangga* (CM) were found to have good stability in physiological media after 24 h of dispersion. It is also cytocompatible with human colon fibroblast cells (CCD-18Co) and human lung fibroblast cells (MRC-5). Hemocompatibility tests revealed that these gold NPs were blood-compatible, with less than 10% of hemolysis without any aggregation of erythrocytes. This study suggests the potential in employing a CM-extract-based method in the preparation of gold nanoparticles for anticancer diagnosis and therapy [200].

A systemic study on the accumulation of *Helianthus tuberosus*-mediated gold NPs in rats revealed that gold element concentration accumulated in the liver, lung, kidney, and spleen. The study showed a that the lung is the major target organ and further suggests that enduring administration could lead to organ damage as majorly observed in lung tissue. This study suggests the necessity of complete in vivo toxicity analysis, before introducing NPs in biomedical applications [201].

At present, there are very few reports of biocompatibility study of biological metal nanoparticles on animal models that have been available [202, 203]. The atomic economy of biological NPs is controlled by the reduction efficiency of plant molecules that affect the number of surface atoms, single or agglomerated form, uncapped or partially capped or fully capped, morphology, and possibility of unreduced metallic ions these all factors, in turn, affects the cytotoxicity of biological NPs during toxicity study of biological NPs. The quick, efficient, economic protocol needs to develop toxicity study NPs [198].

## Other applications of metallic nanoparticles

With the growing awareness in nano-based themes and applications, the nano-based sensor is occupying an eminent role in scientific studies. Metal nanoparticles are preferentially used in the transducer component of sensors; that too silver, gold, and platinum is widely used. Though reports on nanosensors are practiced in glucose detection, immunosensors, aptamers. But here, the green metallic nanoparticles as a nano-sensor are utilized in very few studies only. Alex et al. [204] reported the sensitivity of biologically synthesized silver nanoparticles and compared their sensitivity with others and concluded that biological silver nanoparticles showed high sensitivity which can be employed in various cost-effective and eco-friendly sensor devices applications. They have demonstrated the sensitivity of silver nanoparticles toward MCZ fungicide with the 39.1 nm/mM. In another study, the sensitivity of gold nanoparticles synthesized using *C*. *nudiflora* plant extract was used for the detection of HCG hormone in pregnant women urine with 100% accuracy [205]. Similarly, gold nanoparticles can be employed as a biosensor to determine the glucose content in commercial glucose injections was successfully achieved and performed [206].

## Future research and outlook of metallic nanoparticles

With the inception of NPs over a half-century, the perception of NPs is still now not clearly understood by the researchers. Green chemistry philosophy warrants the synthesis of NPs as an eco-friendly alternative for conventional methods of NPs synthesis. Moreover, the green chemistry approach of NPs synthesis stands on the viewpoint that NPs synthesis should be a benign process, utilization of natural resources, avoiding usage of hazardous materials, free from toxicity and cost inexpensive.

Hitherto, numerous reports have documented the synthesis of metal/metal oxide NPs using the resources plants, bacteria, fungi, yeast, and actinomycetes. Among the natural resources, plants are widely employed for NPs synthesis owing to the ethnobotanical value, active ingredients, easily available, simplified process, and cost inexpensive. Despite the facts, there are a lot of key issues and technical challenges to be addressed by the researchers to develop green NPs as a successful one.

Physical and chemical-based methods of NPs synthesis produce uniformity, homogeneity, and mono NPs but in biological-based, it is questionable.

The following are the key issues about green NPs synthesis and development:

- ➢ Lack of holistic knowledge to develop green NPs using plants entity.
- ➢ The logical strategy should be adopted to develop green NPs with discrete size and shape.
- ➢ Uniformity of NPs should be ensured. Plant-mediated NPs produce more variant size, shape, and structure.
- ➢ Conversion of salt to ion is the main challenge to be addressed. In plant-mediated synthesis, the maximum conversion of salt to ion should be accomplished.
- ➢ The precise role of plant molecules in NPs should be elucidated. These molecules act as a reducing and stabilizing agent.
- ➢ Whether the NPs fabricated are homogenous since there is a difference in substance [biological resources] utilized for synthesis.
- ➢ The transfer of technology processes should be implemented to fabricate NPs from the lab to the industrial level.
- ➢ Industrial production of NPs should have come with a benign method focusing on ease of synthesis, utilization of resources, particle generation [monodispersity, uniformity, reproducibility], waste management, and toxicity perspective.
- ➢ It is a distant dream to produce NPs completely free from toxicity. In our review also, we explain the potential threat of toxicity of plant-mediated metallic nanoparticles to humans and the environment. Henceforth, at least researchers should be directed to fabricate NPs with minimal toxicity.
- ➢ It is also imperative to understand the ecotoxicological perspective of metallic NPs for environmental applications. Studies on the aquatic ecosystem, various habitats on niche areas, nontarget organisms should also be carried out.

Another important and most serious concern to be addressed is the utilization of NPs in biomedical applications. Infectious diseases are caused by bacteria, viruses, fungi, and parasites. In practice, routine usage of antibiotics led to the development of resistance mechanisms by microbes. In some cases, these antibiotics also create toxicity to humans and they are non-selective too.

We are living in an exciting age where these size dependencies offer both challenges and opportunities, and that, if we take the appropriate approach, this will give us more room for discoveries and applications.

## Conclusion

Herein, we have comprehensively provided the recent trends in the synthesis of metallic nanoparticles through plants only. The present review aims to the concept and demands the need for a synthesis of metallic nanoparticles from various plants. Moreover, we strongly focused on the challenges encountered in the synthesis of nanoparticles and characterization convincingly. Further, we advocated the applications of metallic nanoparticles such as antimicrobial, antioxidant, anticancer, anti-inflammatory, wound healing, larvicidal, and leishmanicidal activities of metallic nanoparticles in context with recent findings. Finally, we highlighted the future perspective of metallic nanoparticles with strong recommendations and necessitate the changes to be adopted for developing metallic nanoparticles as a safe biocompatible agent. Overall, considering all the above scientific merits and demerits of metallic nanoparticles, researchers tune their research toward metallic nanoparticles from plants by ease process and develop such kinds of metallic nanoparticles as theranostics for various infectious and noninfectious diseases.

## Abbreviations

NP: Nanoparticles
CN: Carbon nanotubes
MN: Metal nanoparticles
CN: Ceramic nanoparticles
SN: Semiconductor nanoparticles
SLIPS: Slippery liquid-infused porous surface
CVD: Chemical vapor deposition
RPM: Rotation per minute
pH: Potential of hydrogen ion concentration
UV-Vis spectra: Ultraviolet-visible spectroscopy
Au: Gold
HRTEM: High-resolution transmission electron microscopy
MRSA: Methicillin-resistant *Staphylococcus aureus*
VRSA: Vancomycin-resistant *Staphylococcus aureus*
IL: Interleukins
TNF: Tumor necrosis factor
AgNPs: Silver nanoparticles
LPS: Lipopolysaccharide/endotoxin
AuNPs: Gold nanoparticles
ROS: Reactive oxygen species
NF: Necrosis factor
COX: Cytochrome oxidase
kDa: Kilo Dalton
Fe_3_O_4_: Iron oxide
BNC: Bacterial nano cellulose
VEGF: Vascular endothelial growth factor
FGF: Fibroblast growth factor
EGF: Epidermal growth factor
LDH: Lactate dehydrogenase
DDT: Dichlorodiphenyltrichloroethane.
HSV: Herpes simplex virus
HIV: Human immunodeficiency virus
IC: Inhibitory concentration
μg/mL: Microgram/milliliter
ZnO NPs: Zinc oxide nano particles
LC: Lethal concentration
DPPH: 1,1-Diphenyl-2-picrylhydrazyl
MTT: 3[4,5 Dimethylthiozole-2yl]-2,5 diphenyltetrazolium bromide
XTT: 3-bis-[2-Methoxy-4-nitro-5-sulfophenyl]-2H-tetrazolium-5-carboxanilide
ICP-OES: Inductively coupled plasma-optical emission spectrometry
CAT: Catalase
GST: Glutathione S-transferase
GPx: Glutathione peroxidase
